# Tamoxifen accelerates the repair of demyelinated lesions in the central nervous system

**DOI:** 10.1038/srep31599

**Published:** 2016-08-24

**Authors:** Ginez A. Gonzalez, Matthias P. Hofer, Yasir A. Syed, Ana I. Amaral, Jon Rundle, Saifur Rahman, Chao Zhao, Mark R. N. Kotter

**Affiliations:** 1 Anne McLaren Laboratory for Regenerative Medicine, Department of Clinical Neurosciences, Wellcome Trust and MRC Cambridge Stem Cell Institute, University of Cambridge, West Forvie Building, Forvie Site, Robinson Way, Cambridge CB2 0SZ, UK

## Abstract

Enhancing central nervous system (CNS) myelin regeneration is recognized as an important strategy to ameliorate the devastating consequences of demyelinating diseases such as multiple sclerosis. Previous findings have indicated that myelin proteins, which accumulate following demyelination, inhibit remyelination by blocking the differentiation of rat oligodendrocyte progenitor cells (OPCs) via modulation of PKCα. We therefore screened drugs for their potential to overcome this differentiation block. From our screening, tamoxifen emerges as a potent inducer of OPC differentiation *in vitro*. We show that the effects of tamoxifen rely on modulation of the estrogen receptors ERα, ERβ, and GPR30. Furthermore, we demonstrate that administration of tamoxifen to demyelinated rats *in vivo* accelerates remyelination. Tamoxifen is a well-established drug and is thus a promising candidate for a drug to regenerate myelin, as it will not require extensive safety testing. In addition, Tamoxifen plays an important role in biomedical research as an activator of inducible genetic models. Our results highlight the importance of appropriate controls when using such models.

In the central nervous system (CNS), oligodendrocytes extend processes that form highly organized membrane wraps around axons^1^. These so-called myelin sheaths form the structural basis for rapid conduction of nervous impulses^1^ and have important protective and trophic functions^2^,^3^.

Loss of myelin occurs in a number of neurological disorders. In the most prominent demyelinating disease, multiple sclerosis (MS)^4^,^5^,^6^,^7^, myelin sheaths are damaged by an autoimmune process^8^. With the advent of new drugs that slow the progression of the autoimmune disease, the question of how the regeneration of myelin sheaths can be enhanced is receiving increasing attention. However, so far, no remyelination-promoting therapy is available in a clinical setting.

Failure of remyelination can occur as a consequence of insufficient recruitment of oligodendrocyte progenitor cells (OPCs) into demyelinating lesions^9^. Furthermore, intrinsic changes within OPCs and factors that accumulate in MS lesions are able to inhibit OPC differentiation^10^,^11^,^12^,^13^. Lesion-associated inhibitors regulate distinct signaling cascades in OPCs, and it may be possible to manipulate them pharmacologically to promote myelin regeneration. For example, we recently demonstrated that inhibition of Phosphodiesterase (PDE)4 is able to promote CNS remyelination^14^. Other approaches include inhibiting Leucine rich repeat and Immunoglobin-like domain-containing protein (Lingo)-1^15^, Wingless/mouse mammary tumor virus integration site (Wnt) signaling^16^ and Retinoic acid receptor (RXR)-**γ**^17^.

Previous studies, including our own work, demonstrated that myelin proteins, which accumulate following demyelination, are able to inhibit remyelination by blocking the differentiation of oligodendrocyte progenitor cells (OPCs)^18^,^19^,^20^. Investigating the underlying mechanisms we found that myelin proteins inhibit OPC differentiation by modulating RhoA and PKCα signalling^21^,^22^. On the basis of these results, we investigated the potential of PKCα inhibitors to overcome the myelin-associated differentiation block to promote OPC differentiation. We found that tamoxifen promoted this most effectively, albeit via an alternative mechanism.

## Results

**Figure 1:**
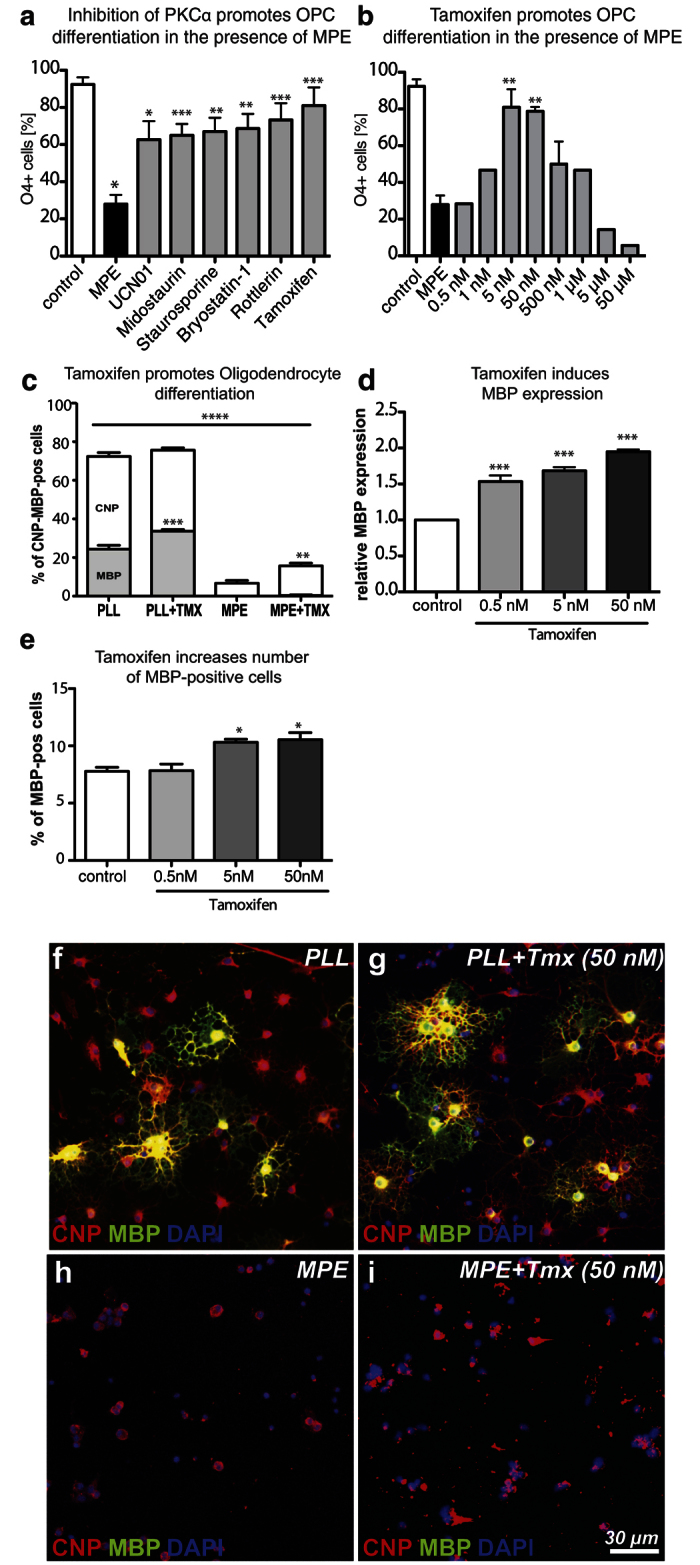
Tamoxifen promotes OPC differentiation in the presence and absence of myelin associated inhibitors. (**a**) Bar graph demonstrating differentiation-promoting effects of PKCα inhibitors (7-hydroxystaurosporine (UCN01): 10 nM, midostaurin: 1 nM, staurosporine: 25 nM, bryostatin-1: 5 nM, rottlerin: 5 nM, and tamoxifen: 5 nM) on OPCs cultured on inhibitory myelin substrates (myelin protein extract, MPE) after 2 days. O4 expression is an early marker of OPC differentiation (n = 4). One-way ANOVA with Dunnett’s post-hoc test: MPE vs UCN01, midostaurin, staurosporine, bryostatin-1, rottlerin, and tamoxifen: *p < 0.05, **p < 0.01, ***p < 0.0001. (**b**) Bar graph evaluating the efficacy of tamoxifen-induced OPC differentiation in the presence of MPE at concentrations ranging from 0.5 nM to 50 μM. (n = 4), One-way ANOVA with Dunnett’s post-hoc test: control 2d vs 5, 50, 500 nM: *p < 0.05, **p < 0.01, ***p < 0.0001. (**c**) Bar graph demonstrating that 5 nM tamoxifen is able to increase the number of MBP-immunopositive cells plated on poly-L-lysine control substrates. In the presence of MPE, tamoxifen is able to increase the number of CNP positive cells after 2 days differentiation. (n = 3); ANOVA: CNP ****p < 0.0001, MBP ****p < 0.0001; Dunnett’s post-hoc test PLL vs PLL + Tmx: MBP ***p < 0.0001; MPE vs. MPE + Tmx: CNP ***p < 0.0001). (**d**) Bar graph showing quantification of *Mbp* expression using qRT-PCR for *Mbp* relative to *Gapdh* mRNA. Tamoxifen increased *Mbp* expression in OPCs plated on PLL control substrates after 2 days in dose-dependent manner. One-way ANOVA with Dunnett’s post-hoc test: control 2d vs 5, 50, 500 nM: ***p < 0.0001. (**e**) Bar graph demonstrating that tamoxifen is also able to increase the number of MBP-positive OPCs plated on poly-L-lysine (PLL) control substrates after 2 days. 1-way ANOVA with Dunnett’s post-hoc test: control 2d vs 5, 50, 500 nM: *p < 0.05. (**f–i**) Representative images of CNP-MBP-positive OPCs treated with vehicle or tamoxifen (50 nM) in presence and absence of MPE. Error bars: standard error of the mean (SEM). Scale bar: in f-i = 30 μm.

Prompted by our previous findings^22^, which showed that OPC-differentiation-inhibiting myelin breakdown products activate protein kinase C (PKC)α signalling, we investigated the potential of PKCα inhibitors to promote OPC differentiation in the presence of myelin protein extracts by assessing the expression of O4 (Fig. 1). Of the drugs tested (UCN01, midostaurin, staurosporine, bryostatin-1, rottlerin, and tamoxifen), tamoxifen showed particularly potent differentiation-inducing effects. Addition of tamoxifen to OPCs plated on control substrates or to cells on myelin substrates increased the number of MBP+ and CNP+ cells respectively (Fig. 1c, e–i). Quantitative RT-PCR assessment of *Mbp* relative to *Gapdh* mRNA expression demonstrated that tamoxifen treatment induced *Mbp* expression on the transcriptional level (Fig. 1d). The increase of MBP+ cells in response to tamoxifen was concentration dependent (Fig. 1e).

### Tamoxifen is known to modulate estrogen receptor signaling and PKCα activity

**Figure 2:**
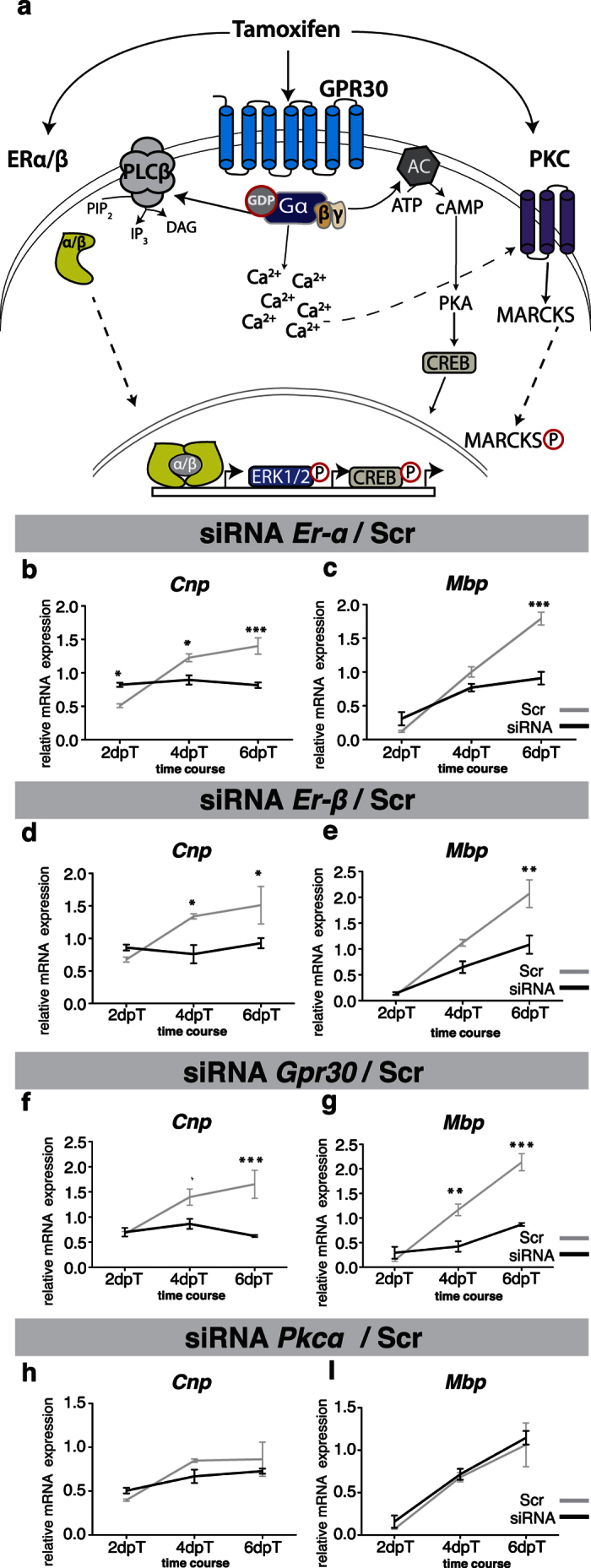
ERα, ERβ, and GPR30 are important regulators of OPC differentiation. (**a**) Model of tamoxifen signalling in OPCs: (1) tamoxifen binds nuclear ERα/β receptors, which respond by modulating transcriptional events; (2) tamoxifen can also activate membrane bound GPR30 receptors, inducing an increase in intracellular Ca2 + levels. This in turn activates adenylate cyclase (AC), resulting in activation of protein kinase A (PKA) and CREB1. Furthermore, (3) tamoxifen is also able to inhibit PKCα, which is known to modulate cytoskeletal proteins, including MARCKS, and inhibit OPC differentiation. (**b–i**) Line graphs demonstrating impaired OPC differentiation (decreased expression of *Cnp* and *Mbp* mRNA relative to glyceraldehyde-3-phosphate dehydrogenase) when OPCs were transfected with siRNA for *Er-α*, *Er-β* or *Gpr30* as compared with non-targeting scrambled siRNA (Scr, gray line). Gene silencing of *Prkcα* did not negatively affect the differentiation of OPCs on control substrates (n = 3). Oneway ANOVA with Bonferroni’s post-hoc tests: *p < 0.05, **p < 0.01, ***p < 0.0001. dpT, days post siRNA transfection. Error bars: SEM.

Tamoxifen has been used for the treatment of breast cancer since the early 1970s^23^. In addition to inhibiting PKCα activity, tamoxifen is known to modulate estrogen signaling (Fig. 2a). So far, two classes of estrogen receptors have been identified: the nuclear estrogen receptors alpha (ERα) and beta (ERβ), and a membrane-bound G-protein-associated estrogen receptor (GPR30). Whereas ERα and ERβ are estrogen-responsive nuclear receptors that act as transcriptional regulators^24^, GPR30 is known to modulate several signaling pathways such as the phosphatidylinositol-3 kinase and mitogen-activated protein kinase pathways^25^.

### Estrogen receptors are positive regulators of OPC differentiation

The literature suggests that all three ER receptor types are expressed in oligodendrocyte lineage cells^26^,^27^,^28^,^29^,^30^,^31^,^32^,^33^,^34^. To investigate whether sex influences ER expression of neonatal rat OPCs were separately derived from female and male pups. We found that *Er-α*, *Er-β*, or *Gpr30* expression was comparable in both genders (Supplementary Fig. 1). Based on these results we continued to use OPCs derived from pooled rat pups including both genders.

An interesting property of tamoxifen is that, depending on the cell type, it can act as agonist or antagonist on estrogen receptors^35^. For example, it has an *agonistic* effect in the uterus and skeleton but *antagonistic* activity in the breast, where it has been used for several years for the treatment of breast cancer.

We first sought to elucidate how ER or PKCα signaling affects the differentiation of OPCs into oligodendrocytes. For this purpose we transfected OPCs with small interfering (si)RNA directed against individual ERs and PKCα (Fig. 2). Individual silencing of *Er-α, Er-β*, or *Gpr30* resulted in decreased OPC differentiation reflected by reduced mRNA expression of the differentiation markers cyclic nucleotide 3′-phosphodiesterase (*Cnp)* and myelin basic protein *(Mbp)* (Fig. 2b–g).

In contrast, gene silencing of PKCα did not alter the differentiation of OPCs on control substrates (Fig. 2). This was expected as with the culture conditions applied, inhibition of PKCα only shows effects in the presence of inhibitory myelin substrates^22^. Taken together these results confirm that ERα, ERβ, and GPR30 have important functional roles during the process of OPC differentiation (see also Supplementary Fig. 3).

### Tamoxifen-induced OPC differentiation depends on the presence of ER

**Figure 3:**
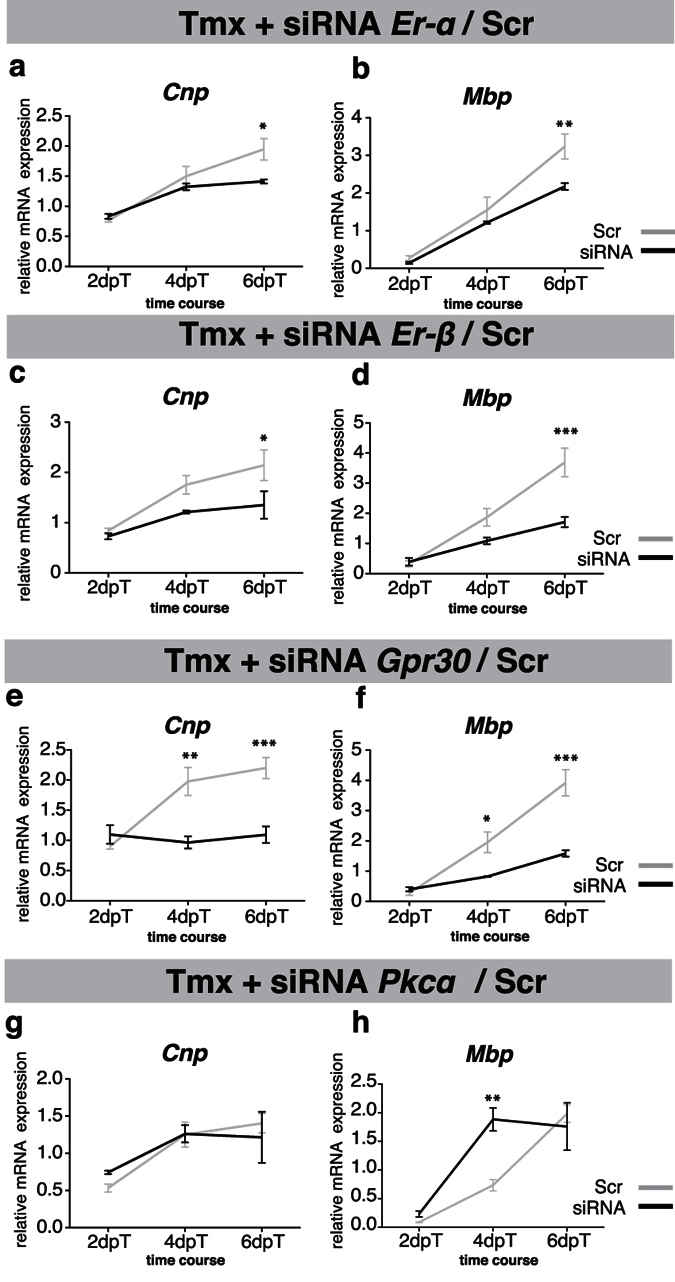
Tamoxifen-induced OPC differentiation depends on ERα, ERβ, and GPR30, but not PKCα signaling. (**a–h**) Line graphs demonstrating that tamoxifen-induced differentiation (+Tmx (50 nM)) of OPCs is impaired when cells are concomitantly transfected with siRNA targeting *Er-α*, *Er-β*, or *Gpr30* as compared with control siRNA (scrambled, Scr, gray line). Gene silencing of *Prkcα* in addition to tamoxifen treatment resulted in transiently increased *Mbp* expression at day 4, demonstrating that tamoxifen-induced OPC differentiation is independent from PKCα (n = 3). One-way ANOVA with Bonferroni’s post-hoc tests: *p < 0.05, **p < 0.01, ***p < 0.001. Error bars: SEM.

To investigate the mechanism by which tamoxifen promotes OPC differentiation, we silenced ERα, ERβ, GPR30, and PKCα in cells treated with tamoxifen. Similar to OPCs cultured in the absence of tamoxifen, silencing of ERα, ERβ, or GPR30 negatively affected the differentiation of cells in the presence of tamoxifen, indicating that the differentiation-inducing effects of tamoxifen are dependent on the presence of each of the ERs tested (Fig. 3a–f and see Supplementary Fig. 4a–h for calculations of the difference in *Cnp* or *Mbp* mRNA expression between “siRNA - tamoxifen” and “siRNA + tamoxifen conditions”).

On the other hand, gene silencing of PKCα in combination with tamoxifen treatment resulted in increased *Mbp* expression at early time points as compared to tamoxifen treatment alone (Fig. 3g,h). Tamoxifen therefore seems to exert OPC-differentiation-inducing effects via ERs but not via PKCα.

### Assessing the effects of tamoxifen in an *in vivo* model of remyelination

We next sought to investigate whether tamoxifen can promote OPC differentiation in a model of CNS remyelination. The largest targeted demyelinating lesions in a rodent model have so far been achieved by injection of ethidium bromide (EB) into white-matter tracts of the rat brainstem. Since the efficiency of remyelination is inversely correlated with lesion size, this model is advantageous for studying the kinetics of CNS remyelination^36^. Application of EB results in rapid and complete depletion of glial cells, including astrocytes and oligodendrocyte lineage cells, within 3 days with good preservation of axons. As a consequence the axons become demyelinated. This is followed by a highly predictable sequence of events during which the lesion becomes fully remyelinated^37^. In young-adult rats, remyelination is complete within 28 days.

### Estrogen receptors and active PKCα are expressed by oligodendrocyte lineage cells *in vivo*

**Figure 4:**
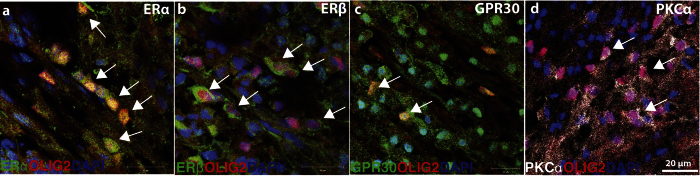
Expression of estrogen receptors and active PKCα at 14 pli. (**a–d**) Representative images taken at 40 × showing expression of estrogen receptors ERα, ERβ and GPR30 (green) and phosphorylated (active) PKCα (white) by OLIG2 + oligodendrocyte lineage cells (red) in CCP lesions 14 days post ethidium bromide injection (pli). White arrows point ERs + or PKCα + /OLIG2 + cells. Scale bar: 20 μm.

To confirm that ERα, ERβ, and GPR30, as well as activated PKCα, are present in remyelinating lesions, we carried out immunohistochemical staining using antibodies directed against the respective antigens and included co-stainings for the oligodendrocyte lineage cell-specific transcription factor OLIG2. This confirmed the presence of all three ERs and active PKCα in oligodendrocyte lineage cells within remyelinating lesions (Fig. 4).

### Tamoxifen induces OPC differentiation in demyelinated lesions

**Figure 5:**
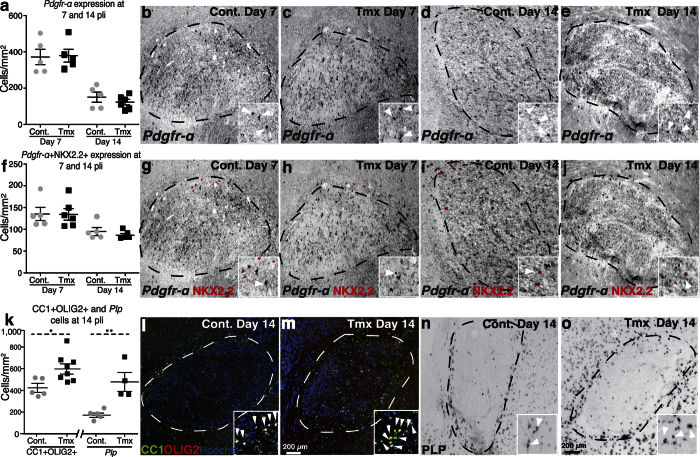
Tamoxifen promotes OPC differentiation after toxin-induced demyelination in white matter. (**a**) Scatter plot showing comparable numbers of immature OPCs expressing *Pdgfr-α* in remyelinating lesions at day 7 and 14 (n ≥ 5), Student’s *t*-test 7 days pli: p = 1.000, 14 days pli: p = 0.2468. (**b–e**) Representative sections of tamoxifen- (Tmx) and vehicle treated animals labeled by *in situ* hybridization for *Pdgfr-α* at 7 pli and 14 pli. (**f**) Scatter plot demonstrating comparable numbers of *Pdgfr-α* + /NKX2.2 + activated OPCs in lesions at day 7 and 14 days after demyelination (n ≥ 5), Student’s *t*-test 7 pli: p = 0.4206, 14 pli: p = 0.9269. (**g,j**) Representative sections labeled by *in situ* hybridization for *Pdgfr-α* and immunohistochemistry for NKX2.2 at 7 pli and 14 pli. (**k**) Scatter plots demonstrating that tamoxifen treatment significantly promotes OPC differentiation at 14 pli on the basis of increased expression of mature markers (CC1 + /OLIG2 + and *Plp* + cells) 14 pli (n ≥ 5), Student’s *t*-test, CC1 + /OLIG2 + : p = 0.0274, *Plp* + : p = 0.0062. (**i–o**) Representative sections of lesions labeled by immunohistochemistry for CC1 and OLIG2, or *in situ* hybridization for *Plp* at 14 pli. White arrows in the insets point to double positive cells. Error bars: SEM. Scale bar: in b-e, g-j, l-o = 200 μm.

To investigate whether tamoxifen treatment is able to induce OPC differentiation *in vivo*, animals receiving daily intraperitoneal injections of tamoxifen at two different doses (0.5 mg/day and 2 mg/day) were compared with controls receiving vehicle injections. Several stage-specific molecular markers were used to characterize the OPC response to tamoxifen in remyelinating lesions. We analyzed lesions 7 days post lesion induction (pli) to assess the recruitment of OPCs and lesions 14 days pli to assess the differentiation of OPCs into mature oligodendrocytes (Fig. 5a–e). Whereas the number of immature *Pdgfrα* OPCs or “activated” NKX2.2-expressing OPCs was comparable between the groups 7 and 14 days pli (Fig. 5f–j), a significant increase in cells expressing the mature oligodendrocyte markers adenomatous polyposis coli (APC(CC1)) and proteolipid protein (*Plp*) was detected in animals receiving the higher treatment dose 14 days pli (Fig. 5k–o). These findings indicate that tamoxifen treatment induced the differentiation of OPCs in the lesions.

However, myelin-sheath formation is also regulated at levels downstream of the transcriptional program. Does the enhanced expression of mature markers therefore correlate with an accelerated formation of new myelin sheaths?

### Tamoxifen promotes CNS remyelination

**Figure 6:**
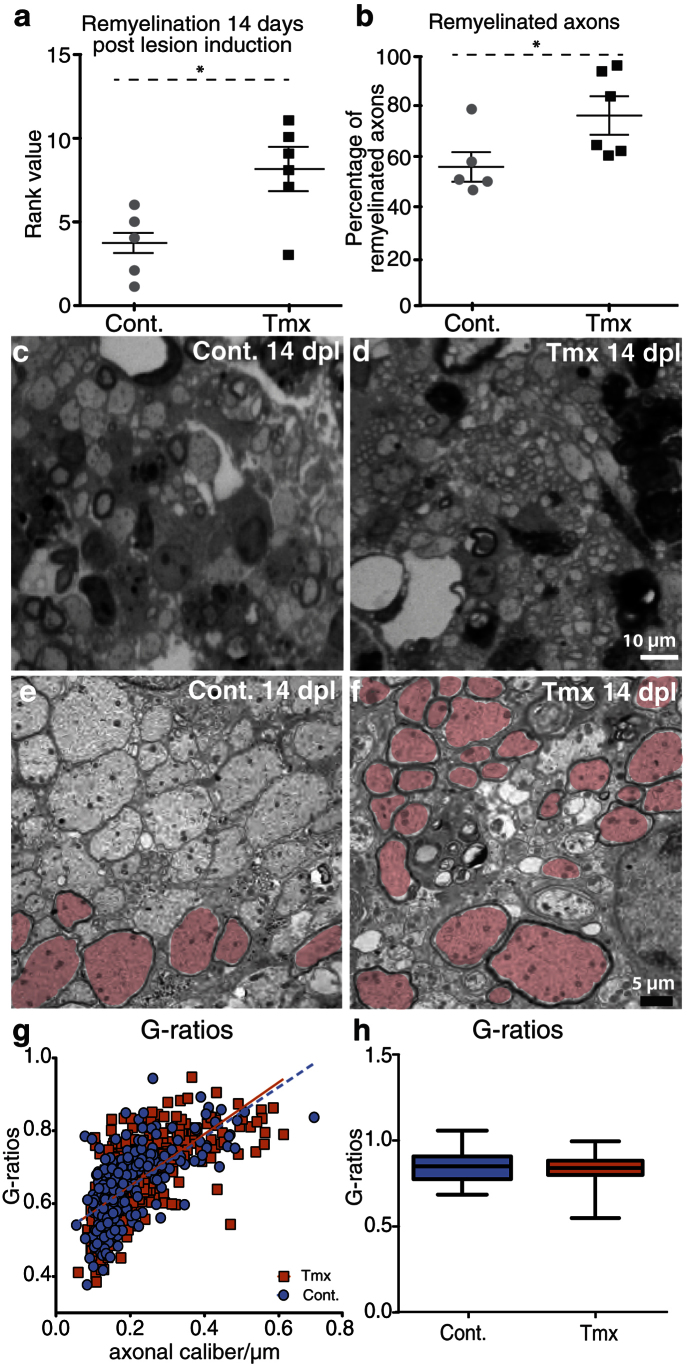
Tamoxifen treatment accelerates CNS remyelination. (**a**) Rank analysis (scatter plot) of remyelination in CCP lesions at 14 pli assessed on toluidine-blue-stained semithin sections. Higher rank scores indicate a greater extent of remyelination (n ≥ 5); Mann-Whitney U test: p < 0.05. (**b**) Enhanced remyelination was confirmed by manual counts of remyelinated and demyelinated axons on digitized sections (n ≥ 5). Mann-Whitney U test: p < 0.05. (**c,d**) Representative toluidine blue-stained semithin sections (100x) of vehicle (‘Cont.’) and tamoxifen-treated animals. (**e,f**) Representative electron micrographs of control and tamoxifen-treated animals (remyelinated axons pseudo-colored). (**g,h**) Scatter plot and box plot of G-ratios demonstrating comparable relative thickness of newly formed myelin sheaths in control and tamoxifen-treated lesions. Student’s *t*-test: p > 0.05. Error bars: SEM. Scale bar: in c, d = 10 μm; in e, f = 5 μm.

To investigate the extent of CNS remyelination at 14 days, the experiments were repeated using the higher dose of tamoxifen and the tissue was embedded into resin for ultrastructural analysis. Investigator-blinded rank analysis demonstrated that tamoxifen significantly increased the number of remyelinated axons (Fig. 6a). This was confirmed by determining the number of remyelinated axons on high-power digitized micrographs of caudal cerebellar peduncle lesions (Fig. 6b). The relative thickness of newly formed myelin did not differ between the groups (Fig. 6c–h). Taken together, these data demonstrate that tamoxifen treatment resulted in accelerated remyelination at 14 days pli.

### Tamoxifen treatment does not alter the macrophage response

Our *in vitro* data indicate that tamoxifen can directly regulate OPC differentiation. However, it is possible that tamoxifen also modulates the function of other cell types in demyelinated lesions. In particular, macrophages are important determinants for the efficiency of remyelination^38^,^39^. We initially assessed the number of osteopontin-positive macrophages in the lesions. No differences were detected between the groups with respect to the overall macrophage presence (Supplementary Fig. 5). Recent findings highlight the importance of macrophage activation, specifically of M1 and M2 phenotypes^40^. Quantification of the presence of CCR7^+^-M1 and Arginase1^+^-M2 macrophages 7 days post lesion induction did not show significant differences between the tamoxifen treated and control animals. Furthermore, assessment of phagocytic activity in remyelinating lesions by staining intracellular myelin-derived neutral lipids with Oil Red-O did not detect differences between the groups. Nevertheless, further indirect effects of tamoxifen cannot be ruled out entirely.

## Discussion

Here we have shown that tamoxifen induces OPC differentiation and remyelination *in vitro* and *in vivo*, and that this relies on modulation of the estrogen receptors ERα, ERβ, and GPR30. The effects of tamoxifen do not involve the PKCα pathway.

Estrogen receptors play an important role in OPC differentiation and remyelination^31^,^32^,^33^,^41^. Similarly, in our experiments, silencing of ER resulted in impaired OPC differentiation *in vitro*, even estrogen was not specifically added to the medium. However, fetal calf serum (FCS) used in the OPC media does contain several components, which could activate estrogen receptors in a ligand-independent manner. Picard 2003, describes a list of molecules which could cross-talk with the estrogen receptors without physically interacting with them, for example, insulin (which forms an important component of the differentiation medium), IGF and FGF^42^. Moreover, testosterone (present in FCS) could be converted by aromatase, which is expressed in low levels in glial cells (http://web.stanford.edu/group/barres_lab/brain_rnaseq.html), into estrogen and therefore, activate all estrogen receptors. As a results, silencing of ER is likely to impair OPC differentiation in our culture model.

When estrogen receptors were silenced, the differentiation-inducing effects of tamoxifen were reduced, indicating an involvement of ER (or GPR30) in tamoxifen-mediated effects. In contrast, tamoxifen treatment in combination with silencing of PKCα did not reduce the efficiency of tamoxifen-induced OPC differentiation. In fact we observed transiently increased MBP expression at day 4 compared with tamoxifen treatment alone. While this is only a transient finding at a single time point, it may indicate that combinatorial treatment of tamoxifen and inhibition of PKCα may act synergistically to accelerate OPC differentiation. Nonetheless it can be concluded that differentiation-inducing effects of tamoxifen do not rely on inhibition of PKCα.

Our results also confirm previous reports^22^ that the inhibition of PKCs does not promote differentiation by itself when cells are cultured in serum containing media. Conversely, in the presence of myelin debris, the inhibition of PKCα neutralizes myelin-associated inhibitors and promotes differentiation^22^.

The majority of the reported pharmacological (metabolism and toxicology) studies of tamoxifen use the free base form of tamoxifen and not the soluble forms (citrate tamoxifen and/or 4-OH tamoxifen). Therefore, the present *in vitro* and *in vivo* experiments were also conducted with tamoxifen in its free base form. However, a recent paper by Barrat *et al.* evaluated metabolites of tamoxifen (4-hydroxytamoxifen and endoxifen) as well as tamoxifen citrate, a water-soluble version on a rat glia restricted precursor cell line^43^. They found that all metabolites promoted the expression of late stage markers of OPC differentiation, such as *Cnp* and *Mbp*, which is consistent with our findings using primary rat OPC cultures. In addition, the effect of tamoxifen was blocked when a pan-antagonist was added to the media, supporting our finding that tamoxifen acts as an estrogen receptor agonist in OPC differentiation.

Apart from oligodendroglial cells, tamoxifen potentially also acts on other cell types. We therefore assessed activated macrophages/microglia, which are known to play a key role in remyelination. We did not observe changes in the number of recruited cells or the myelin debris removal at 7 and 14 pli. Furthermore, no differences were observed regarding the number of the pro-inflamatory (M1) or anti-inflammatory (M2) macrophages. Nevertheless, these results cannot entirely rule out potential indirect effects such as differential release of cytokines or chemokines mediated by tamoxifen. The role of astrocytes was not evaluated since their role during remyelination is less clear.

To limit the biological variation and to increase the challenge of the experimental treatment by using a paradigm of efficient remyelination, only young adult female rats were included in the present *in vivo* experiments. The literature indicates that sex-related differences exist with respect to the efficiency of remyelination: whilst in young rats the rate of remyelination is comparable between the sexes, it is more efficient in aged female rats as compared to male rats^44^. Estrogen signalling plays a distinctive role in females. However, investigating OPCs derived from segregated female and male pups with respect to expression of estrogen receptors and their response to tamoxifen did not show any sex-related differences. Nevertheless, it cannot be ruled out that indirect systemic effects exist and that male rats may react differently to tamoxifen treatment. The main reasons why we decided to use female rats in our study were comparability to previous studies and the fact that MS often affects female patients.

In a clinical setting for the treatment of breast cancer tamoxifen was used at doses ranging from 20–40 mg per day. With an estimated average weight of females at 65 Kg, this translates into 0.3–0.6 mg/Kg. The estimated human equivalent in our experiments is 0.27 mg/Kg, which is in keeping with current treatment paradigms in human.

Steroid hormones are known to influence the course of MS and also to modulate demyelination in animal models. During pregnancy, a reduction in the frequency of relapse rate in patients with relapsing–remitting MS (RRMS) has been reported^45^,^46^,^47^. Similarly, a decrease in the severity of the symptoms in comparison with a nonpregnant control group was detected in pregnant mice affected by experimental autoimmune encephalomyelitis, a model of the autoimmune destruction of myelin sheaths occurring in MS^48^. Interestingly, the administration of estriol to nonpregnant women with RRMS showed a reduction in the number of Th1 cells, which are involved in the autoimmune response, and also in the clinical score of the disease^49^. Furthermore, there is evidence that steroid hormones promote myelin repair: administration of progesterone to cerebellar slices after lysolecithin-induced demyelination showed an increase in remyelination^50^ and recently, the same group has shown that administration of progesterone promotes myelin regeneration in chronic demyelinated lesions in the corpus callosum of mice after being fed with cuprizone^51^. These data support the positive effects of steroid hormones and their potential for the treatment of demyelinating diseases.

Considerable efforts are currently being made to develop remyelination-enhancing drugs. The use of tamoxifen has several advantages over the development of new drugs. For example, it has an excellent safety profile that has undergone the test of time by exhaustive application in the clinic. In our study, tamoxifen emerges as a good candidate for testing in an experimental medicine study specifically designed to assess remyelination in MS patients.

Our findings also have important implications for basic research: experiments investigating the function of specific molecules in the CNS on the basis of the widely used CreERT2Lox system rely on the tamoxifen-dependent activation of Cre recombinase for the induction of genetic changes. Our data demonstrate that administration of tamoxifen has a strong influence on CNS stem/precursor cells. To rule out any unintended effects of tamoxifen on the increasingly used inducible CreERT2-rat transgenic models, and potentially also for CreERT2 mice models, the inclusion of adequate controls is advised.

## Methods

### Preparation of primary OPC cultures

Primary OPC cultures were isolated from neonatal Sprague Dawley (postnatal day 0–3) rat forebrains following a standard protocol^20^,^22^ Neonatal pups were euthanized according to “Schedule 1” regulations from the Home Office Animal Procedures Committee UK. Sex-segregated cultures were prepared from female and male only pups. The male-derived OPCs was confirmed by genotyping for the presence of the SRY gene, present only in the Y chromosome (data not shown). Differentiation was induced using Sato’s medium supplemented with 0.5% fetal calf serum (FCS). For all *in vitro* experiments, only cultures with ≥94% purity were used.

### Preparation of myelin-membrane substrates and myelin-protein extracts

Myelin was purified following two rounds of centrifugation using a discontinuous density gradient of sucrose and osmotic disintegration as described previously^20^,^52^. After centrifugation for 1 h at 100,000 × *g*, the interface between the two sucrose solutions was collected, washed with water and pelleted again at 55,000 × *g* for 10 min. The pellet was dissolved in water and incubated for 1 h on ice to introduce an osmotic shock. The solution was then centrifuged again at 55,000 × *g* for 10 min. The resulting pellet was purified by repetition of the sucrose gradient step described above. The myelin-containing interface was collected and, after two washing steps, a pure myelin pellet was obtained and frozen at −80 °C. The frozen myelin pellets were thawed and resuspended in 1% *N*-octyl-β-D-glucopyranoside, 0.2 M Na_3_PO_4_, pH 6.8, 0.1 M Na_2_SO_4_, and 1 mM EDTA, and incubated at 23 °C for 2 h. Following an ultracentrifugation step (100,000 × *g*, for 30 min at room temperature) the supernatants (which are myelin protein extract, MPE) were collected and stored at 80 °C until further use. To test OPC differentiation, slides were coated with MPE following application of poly-l-lysine (PLL) to promote cell attachment to the well.

### Small molecule experiments in OPC cultures

Estrogen receptors’ agonist/antagonist as well as PKC inhibitors (Tocris Bioscience, Bristol, UK) were dissolved in DMSO, water or ethanol as stated in the according data sheet and added to Sato’s differentiation medium in concentrations stated. Dissolved small molecule stock solutions were diluted at least by 1:10000 when added to the cells eliminating eventual side effect of the dissolving agent. Concentrations of the PKC inhibitors were determined in independent dose-finding experiments. Concentrations used for the experiments included in the present study were as followed, 7-hydroxystaurosporine (UCN01): 10 nM, midostaurin: 1 nM, staurosporine: 25 nM, bryostatin-1: 5 nM, rottlerin: 5 nM and tamoxifen: 50 nM.

### Immunocytochemical analysis *in vitro*

OPCs were seeded onto eight-well chamber slides (2 × 10^4^ cells/well) coated with PLL alone or PLL plus MPE. Following 2 days of differentiation, cells were stained with anti-O4 (1:100; Millipore Corporation, Billerica, MA) and anti-MBP (1:300; Millipore Corporation) antibodies. Secondary antibodies conjugated with Alexa 488 and Alexa 555 were used to visualize positive cells (1:300; Invitrogen, Carlsbad, CA)^22^. To assess OPC differentiation, the percentage of O4- or MBP-positive cells relative to >100 DAPI-stained nuclei per experiment for each condition and each experiment in randomly selected eye fields was determined, with the investigator being blind to treatment group. To assess expression of estrogen receptors in male and female OPCs, OPCs isolated from sex-segregated mixed glia cultures were seeded on PLL-coated glass coverslips on 24-well plates (7 × 10^4^ cells/well). Following 2 and 4 days of differentiation, cells were stained with anti-ERα (1:300; Abcam), anti-ERβ (1:300; Abcam), anti-GPR30 (1:300; Invitrogen) and anti-OLIG2 (1:1000; Millipore) or anti-O4 (1:200; SIGMA), followed by staining with the secondary antibodies indicated above. The percentage of ERα, ERβ or GPR30-positive cells relative to >100 oligodendrocyte lineage cells (O4-positive or OLIG2-positive) per experiment for each condition was determined in randomly selected eye fields. Following immunocytochemistry, cells were mounted with Prolong gold antifade mounting medium (Invitrogen). Cells were visualized and digitized at ambient temperature on an LSM 700 confocal microscope (Zeiss, Thornwood, NJ) at 20× and 40× magnification using Zen Application software (Zeiss).

### RNA extraction and quantitative reverse transcriptase polymerase chain reaction (qRT-PCR)

A minimum of 50,000 oligodendrocytes were washed twice with ice-cold phosphate-buffered saline (PBS). OPCs were lysed, and total RNA was extracted and purified using the RNAeasy Mini kit (Qiagen, Hilden, Germany) according to the manufacturer’s protocol. qRT-PCR was conducted as previously described on an Applied Biosystems 7500HT Fast Real-time PCR system^22^ (Applied Biosystems, Foster City, CA). Values are represented as target gene/glyceraldehyde-3-phosphate dehydrogenase (*Gapdh*) ratios. Triplicate measurements were made on three biological replicates.

### siRNA transfections

OPC cultures were kept overnight in Sato’s proliferation medium without antibiotic supplement. On the following day, siRNA smart pool (for details of siRNAs used see Supplementary Table 1) and lipofectamine RNAiMAX were dissolved separately in OptiMem medium (LifeTech, Gaithersburg, MD). Lyophilized ON TARGET plus smart pool siRNA (Dharmacon/ThermoScientific, Waltham, MA) was dissolved in 5 × RNAi buffer, divided into aliquots, and stored at −20 °C. OPCs were transfected using lipofectamine RNAiMAX (Invitrogen) according to the manufacturer’s instructions and using a 1:400 dilution of lipofectamine and 50 nM of siRNA smart pool per reaction. To determine the silencing efficiencies, cell samples were collected at 48 h and 72 h after transfection for RNA extraction (Supplementary Fig. 2).

### Induction of focal demyelination

All experiments were conducted in accordance with the UK Home Office’s animal-welfare regulations and institutional guidelines for animal care and handling and approved to be performed by UK Home Office’s and University Biomedical Support Service under project license number: 70/7715, formerly 80/2228. Female Sprague Dawley rats in estrum (200–220 g) were anesthetized using a combination of 2% isoflurane in oxygen (as carrier gas) and a subcutaneous injection of buprenorphine hydrochloride 0.03 mg/kg (Vetergesic, Animalcare Ltd, Hull, UK). Demyelinated lesions were induced bilaterally by stereotactic injection of EB (0.01%, 4 ml) into the caudal cerebellar peduncle, −10.4 mm caudal, ±2.6 mm lateral, and −7.3 mm ventral from bregma^36^,^37^. Tamoxifen (Sigma-Aldrich, St. Louis, MO) was dissolved in 100% ethanol and diluted with corn oil (Sigma-Aldrich) to a final concentration of 10% ethanol. The treatment animals were injected intraperitoneally with tamoxifen equivalent to 0.5 mg/Kg/day or 2 mg/Kg/day, and the control group received an equivalent volume of the vehicle. In both groups, drug and vehicle were injected on the third day post-lesion (dpl) for 11 days until 14th day post-lesion. Animals were sacrificed after 7 and 14 dpl. To rule out any effects attributable to the size of the lesion, the density of *Plp*-positive cells was assessed in PBS-treated animals by linear regression (GraphPad, Prism, San Diego, CA). The inclusion of lesions smaller than 0.2 mm^2^, which were strongly populated with *Plp*-positive cells, resulted in a significant non-zero slope indicating an effect of the lesion size on the cell density, as shown in previous studies^14^. This may be explained by the fact that in small lesions, recruitment of OPCs and the clearance of myelin debris are more efficient, and thus remyelination becomes more efficient than with larger lesions. No significant correlation between the size of the lesion and the density of *Plp*-positive cells was observed in lesions larger than 0.2 mm^2^, and so only lesions larger than 0.2 mm^2^ were included in the analysis.

### *In situ* hybridization

The expression of a number of marker mRNA species in demyelinated lesions was examined by *in situ* hybridization with digoxigenin-labeled cRNA probes; *Pdgfr-α*, *Plp*, and osteopontin were kindly provided by Dr. Chao Zhao, University of Cambridge. Animals were perfused with 4% paraformaldehyde (PFA) via the left ventricle. The tissue was extracted, postfixed in 4% PFA overnight at 4 °C, and cryoprotected in 20% sucrose. *In situ* hybridizations were carried out on cryostat sections (12 μm) using established protocols^19^,^53^. ImageJ 1.44 (Wayne Rasband, National Institutes of Health, Bethesda, MD) was used to determine the number of positive cells within the lesions on digitized sections. All analyses were conducted with the investigator blind to the treatment group.

### Immunohistochemical analysis *in vivo*

Immunohistochemistry was performed on 12-μm-thick sections of 4% PFA-fixed tissue. Antigen was retrieved using 0.01 M citrate buffer (Dako) at pH 6.0 for 10 min in a 95 °C water bath, and slides were rinsed in PBS (pH 7.4) thee times (10 min each at room temperature). Then, sections were incubated with the blocking solution containing 5% inactivated normal donkey serum (Abcam, Cambridge, MA) and Triton X-100 (0.1%) in PBS for 60 min at room temperature. The following antibodies were stained: anti-OLIG2 (1:300; Millipore), anti-APC(CC1) (1:300; Calbiochem, La Jolla, CA), anti-NKX2.2 (1:300; DHSB), anti-ERα (1:300; Abcam), anti-ERβ (1:300; Abcam), anti-GPR30 (1:300; Invitrogen), and anti-PKCα-p (1:300; Santa Cruz Biotech, Santa Cruz, CA). Sections were incubated with primary antibody overnight, washed three times in PBS, and then incubated with appropriate Alexa 488- or 594-conjugated secondary antibodies (Invitrogen) for 3 h at room temperature. They were then counterstained with DAPI (Sigma-Aldrich). Prolong gold antifade mounting medium (Invitrogen) was used to mount the slides. Slides were examined in a fluorescence microscope (Nikon TE-2000; Nikon, Düsseldorf, Germany), and images were taken by confocal microscopy (Zeiss LSM-700). All quantifications were conducted with the investigators blind to the treatment group. Data are given as mean ± SEM and statistically analyzed. Three sections per marker per animal were analyzed, and a minimum of four animals were used.

### Oil Red-O staining

Sections from control and treatment group were left to dry at room temperature and then placed in a Coplin jar containing propylene glycol (100%) for 5 min. Prewarmed Oil Red-O solution (0.5% in propylene glycol, Sigma-Aldrich) was stained at 65 °C for 10 min. Following incubation for 5 min with differentiation solution (85% propylene glycol), slides were rinsed twice with water and then mounted using a jelly-based mounting media. Representative images of Oil Red-O stained lesions were digitized, and, using ImageJ software, the intensity of the lesion area was measured from the red channel. Data are given as mean ± SEM and statistically analyzed. Three sections per marker per animal were analyzed, and a minimum of four animals were used.

### Histological analysis of remyelination

To assess the extent of remyelination, the tissue was fixed in 4% glutaraldehyde, osmicated, and processed into resin (TAAB Laboratories Equipment Ltd, Reading, UK)^29^. Sections (1 mm) were stained with 1% toluidine blue. The extent of remyelination was then assessed by light and electron microscopy (see below). Based on the thickness (or absence) of myelin sheaths, demyelinated axons, axons bearing native myelin sheaths, and remyelinated axons on light and electron micrographs can unambiguously be distinguished^54^. Lesions were ranked according to the extent of remyelination by two blinded investigators and statistically analyzed using a two-tailed Mann–Whitney test. Increased remyelination corresponds to a higher rank value. In addition, demyelinated versus remyelinated axons were manually counted on 100× digitized pictures randomly taken from the lesion border. Ratios of remyelinated and demyelinated versus the total number of axons were analyzed using Student’s *t*-test.

### Electron microscopy

Ultra-thin sections (1 μm) containing the lesions were stained with aqueous 4% uranylacetate and lead citrate. The sections were visualized on an electron microscope (Hitachi H-600 electron microscope; Hitachi, Tokyo). A minimum of five micrographs per lesion were analyzed from the border of the lesion. The G-ratios (the ratio of axon circumference to myelin circumference) of oligodendrocyte-remyelinated axons were calculated using ImageJ and statistically analyzed (lower G-ratios indicate thicker myelin sheaths) using Student’s *t*-test.

### Statistical analysis

Data were analyzed using GraphPad software (Prism). Student’s *t*-test was performed for single-group comparisons (control vs treatment), and multiple-group comparisons were conducted using one-way ANOVA followed by Dunnett’s post-test. qRT-PCR data were analyzed using one-way ANOVA followed by Bonferroni’s post-test. For rank analysis, a two-tailed nonparametric Mann–Whitney *U* test was used.

## Additional Information

**How to cite this article**: Gonzalez, G. A. *et al.* Tamoxifen accelerates the repair of demyelinated lesions in the central nervous system. *Sci. Rep.* **6**, 31599; doi: 10.1038/srep31599 (2016).

## Supplementary Material

Supplementary Information
