# Supplementary material for: Tamoxifen accelerates the repair of demyelinated lesions in the central nervous system
Source: Sci Rep. 2016 Aug 24;6:31599. doi: 10.1038/srep31599 (PMC4995517; doi:10.1038/srep31599)

Supplementary Information.

Tamoxifen accelerates the repair of demyelinated lesions in the central nervous system

Ginez A Gonzalez+, Matthias P Hofer+, Yasir A Syed, Ana I Amaral, Jon Rundle, Saifur Rahman, Chao Zhao & Mark RN Kotter*

+ These authors contributed equally to this work.

* To whom correspondence should be addressed

Author affiliation:

Anne McLaren Laboratory for Regenerative Medicine, Department of Clinical Neurosciences, Wellcome Trust and MRC Cambridge Stem Cell Institute, University of Cambridge, West Forvie Building, Forvie Site, Robinson Way, Cambridge CB2 0SZ, UK.
Email: mrk25@cam.ac.uk
Tel: +44 1223 747476
Fax: +44 1223 763350

Supplementary Figures.

Supplementary Figure 1

Sex independent expression of ERα, ERβ and GPR30

(a) Bar graphs demonstrating comparable expression of ERα and ERβ mRNA after 2 days of differentiation in OPCs derived from female and male pups. ERα (n = 6): Student's *t*-test: p = 0.785; ERβ (n = 6): Student's *t*-test: p = 0.370 **(b,c)** Quantification of OLIG2-positive cells co-expressing ERα or ERβ at 2 and 4 days of differentiation derived from sex-segregated pups. ERα 2d (n = 3): Student's *t*-test: p = 1.000; ERβ 2d (n = 3): Student's *t*-test: p = 0.678: ERα 4d (n = 3): Student's *t*-test: *p* = 0.116; ERβ 4d (n = 3): Student's *t*-test: p = 0.423. **(d-k)** Representative confocal images of OLIG2+/ERα+ and OLIG2+/ERβ+ cells in male and female cultures at 2 and 4 days of differentiation. **(l)** Bar graphs demonstrating comparable expression of GPR30 mRNA after 2 days of differentiation in male and female oligodendrocytes, (n = 6): Student's *t*-test: p = 0.920. **(m,n)** Quantification of O4-positive cells (male and female cells) co-expressing GPR30 at 2 and 4 days of differentiation, (n = 3): Student's *t*-test: *p* = 0.119 **(o-r)** Representative images of O4+/GPR30+ cells in male and female cultures at 2 and 4 days of differentiation. Error bars: SEM. Scale bar=25 *μ*m.

Supplementary Figure 2

Knockdown efficiencies of siRNA experiments for *Er-α*, *Er-β*, *Gpr30*, and *Prkca*.

(**a**–**d**) Bar graph demonstrating knockdown efficiency on OPCs cultured after 2, 4, and 6 days post-transfection with siRNA for *Er-α*, *Er-β*, *Gpr30*, and *Prkca*. Relative mRNA expression is expressed as the ratio between the gene of interest and *Gadph* (n = 3). One-way ANOVA with Bonferroni's post-hoc test Scr vs 2, 4, and 6 dpT: **p* < 0.05, ***p* < 0.01, ****p* < 0.001, ****p* < 0.0001.

Supplementary Figure 3

Pharmacological activation of ERα, ERβ and GPR30 promotes OPC differentiation.

(**a**–**h**) Bar graphs demonstrating changes in OPC differentiation (relative mRNA expression
of *Cnp* and *Mbp*) after 2 days in differentiation medium on control (PLL) slides using three different doses of: (**a, b**) β-estradiol, a pan-ERα/ERβ/GPR30 agonist (20 nM); (**c, d**) MPP, a selective ERα antagonist (2 nM); (**e, f**) PHTPP, a selective ERβ antagonist (2 nM); (**g, h**) G15, a selective GPR30 antagonist (2000 nM). Three different dose were tested but only the one with the greatest effect is shown. (n = 3). Student's *t*-test: *p < 0.05, **p < 0.01.

Supplementary Figure 4

Tamoxifen promotes OPC differentiation by stimulating ER but not inhibiting PKCα.

Proliferating cells were transfected with siRNA against (**a**) *Er-α*, (**b**) *Er-β*, (**c**) *Gpr30*, and (**d**) *Prkca* and differentiated with addition of 5 nM tamoxifen. Control cells were treated in the same way using non-targeting scrambled (Scr) siRNA. The effect of tamoxifen was

measured by qRT-PCR for *Cnp* and *Mbp*. The effect of oligodendrocytes' *Cnp* and *Mbp* transcription in response to tamoxifen was determined by calculating the difference in *Cnp* or *Mbp* mRNA expression between "siRNA - tamoxifen" and "siRNA + tamoxifen" conditions. Gray bars = ΔTmx Scr. Black bars = ΔTmx siRNA ($n = 3$). Student's *t*-test, * $p < 0.05$, **$p < 0.01$, ***$p < 0.0001$. Error bars: SEM.

Supplementary Figure 5

Tamoxifen does not affect the macrophage response and phagocytic activity of myelin debris.

(**a**) Scatter plot representing the number of osteopontin-positive cells per mm^2 at 7 and 14 pli ($n \geq 5$). Student's *t*-test at 7 pli: p = 1.000, at 14 pli: p = 0.548. (**b**–**e**) Representative sections labeled by *in situ* hybridization for osteopontin at 7 pli (**b,c**) and 14 pli (**d,e**). (**f**) Scatter plot representing the intensity (arbitrary units, a.u.) of Oil Red-O staining per mm^2 at 7 and 14 pli ($n \geq 5$). Student's *t*-test at 7 pli: p = 0.056, at 14 pli: p = 0.413. (**g**–**j**) Representative figures of oil red-O stained lesions from control and tamoxifen-treated group at 7 pli (**g, h**) and 14 pli (**i, j**). Macrophages/microglia can take up M1 and M2 phenotypes. (**k**) Scatter plot representing the number of $CCR7^+$-M1 macrophages per mm^2 14 pli ($n \geq 5$). Student's *t*-test: p = 0.274. (**l,m**) Representative sections labeled by immunohistochemistry for at 14 pli. (**n**) Scatter plot representing the number of $Arginase1^+$-M2 macrophages per mm^2 at 14 pli ($n \geq 5$). Student's *t*-test: p = 0.546. (**o,p**) Representative figures of $Arginase1^+$ at 14 pli. Error bars: SEM. Scale bar=100 μm.

Suppl. Table 1 - siRNA

Product	Gene	Catalogue number	Web page
ON TARGET plus smart pool	Esr1<br>Estrogen receptor alpha	L-091219-02<br>Dharmacon-GE healthcare	http://dharmacon.gelifesciences.com/sirna/on-targetplus-sirna-reagents-rat/?term=ESR1&sourceId=EG/2099&productId=471170D9-5A00-46B5-87FE-A7B9550EF092
ON TARGET plus smart pool	Esr2<br>Estrogen receptor beta	L-097837-02<br>Dharmacon-GE healthcare	http://dharmacon.gelifesciences.com/sirna/on-targetplus-sirna-reagents-rat/?term=ESR2&sourceId=EG/2100&productId=471170D9-5A00-46B5-87FE-A7B9550EF092
ON TARGET plus smart pool	Gper30<br>G protein coupled receptor 30	L-093123-02<br>Dharmacon-GE healthcare	http://dharmacon.gelifesciences.com/sirna/on-targetplus-sirna-reagents-rat/?term=GPER1&sourceId=EG/2852&productId=471170D9-5A00-46B5-87FE-A7B9550EF092
ON TARGET plus smart pool	non-targeting control	D-001810-10<br>Dharmacon-GE healthcare	http://dharmacon.gelifesciences.com/sirna/on-targetplus-non-targeting-control-pool/

Suppl. Table 2 - Antibodies Used

Antibodies	Company	Species	Application
CNP	Abcam (ab6319)	Mouse	Immunocytochemistry
MBP	Millipore (MAB386)	Rat	Immunocytochemistry
ERα	Abcam (ab16460)	Rabbit	Immunohistochemistry<br>Immunohistochemistry
ERβ	Abcam (ab3576)	Rabbit	Immunohistochemistry<br>Immunohistochemistry
GPR30	Invitrogen (PA5-28647)	Rabbit	Immunohistochemistry
PKCα	Santa Cruz (sc-208)	Rabbit	Immunohistochemistry
OLIG2	Millipore (MABN50)	Mouse	Immunocytochemistry<br>Immunohistochemistry
CC7	Abcam (ab126786)	Rabbit	Immunohistochemistry
Arg1	Abcam (ab60176)	Rabbit	Immunohistochemistry
CC1	Abcam (ab16794)	mouse	Immunohistochemistry
NKX2.2	Developmental Studies Hybridoma Bank (74.5A5)	mouse	Immunohistochemistry
O4	Sigma (O7139)	mouse	Immunocytochemistry

Suppl Figure 1

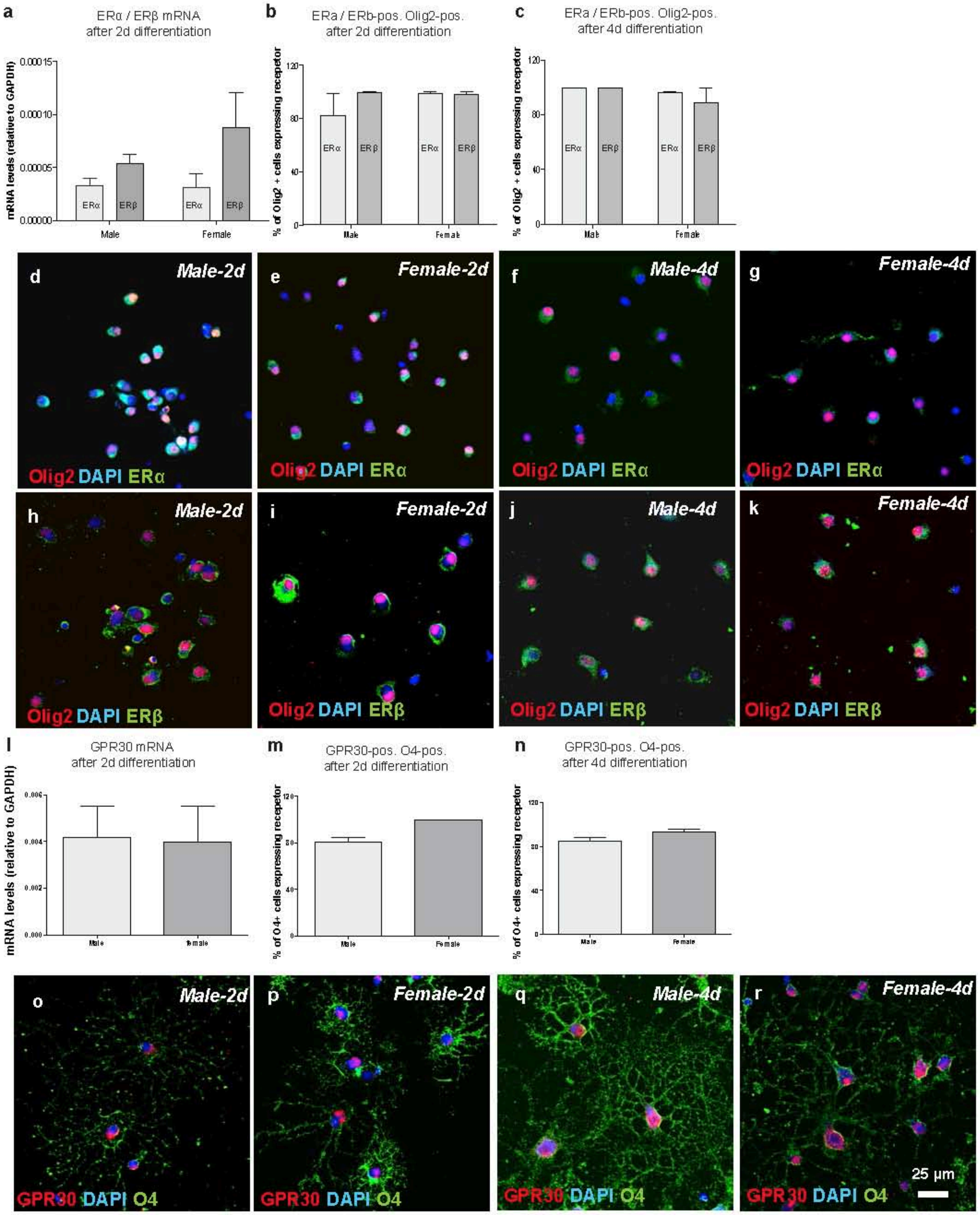

Supplemetary Fig-2

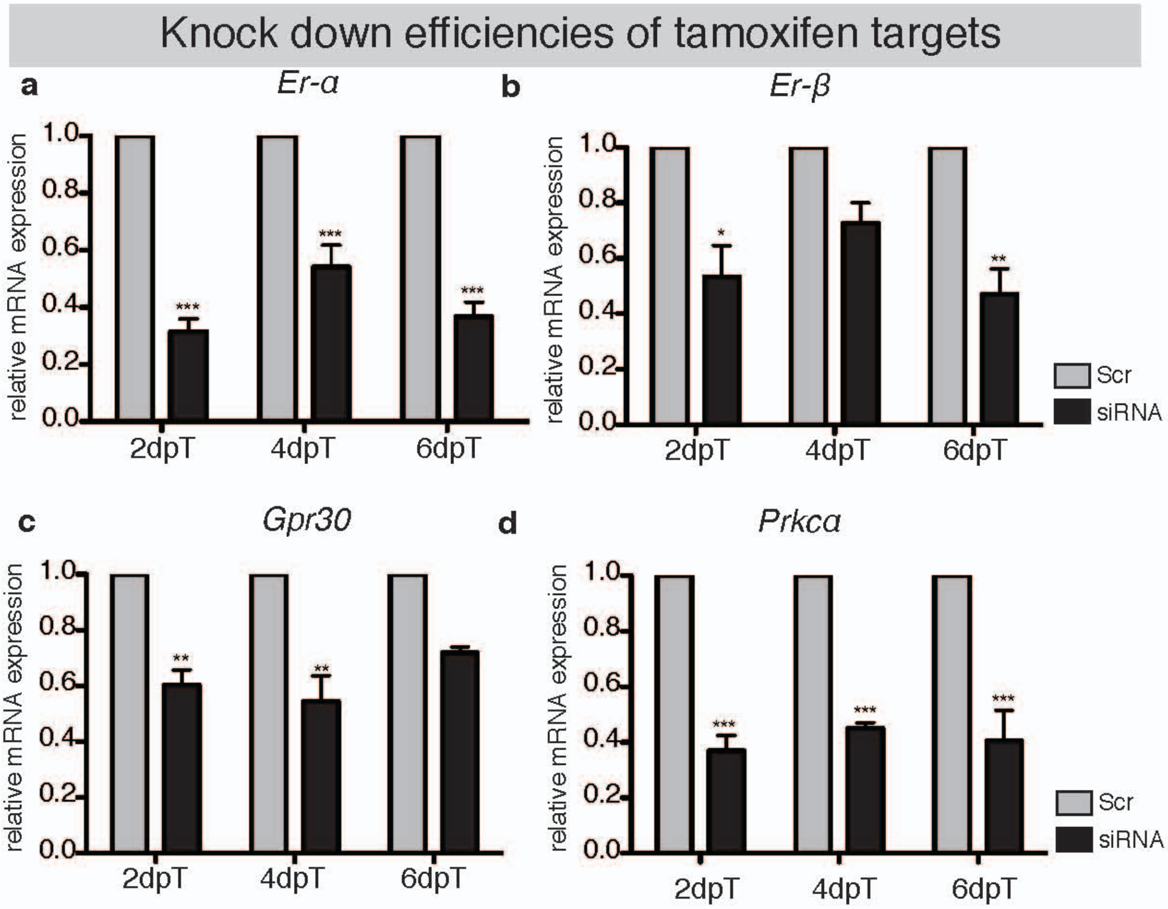

Suppl. Figure 3

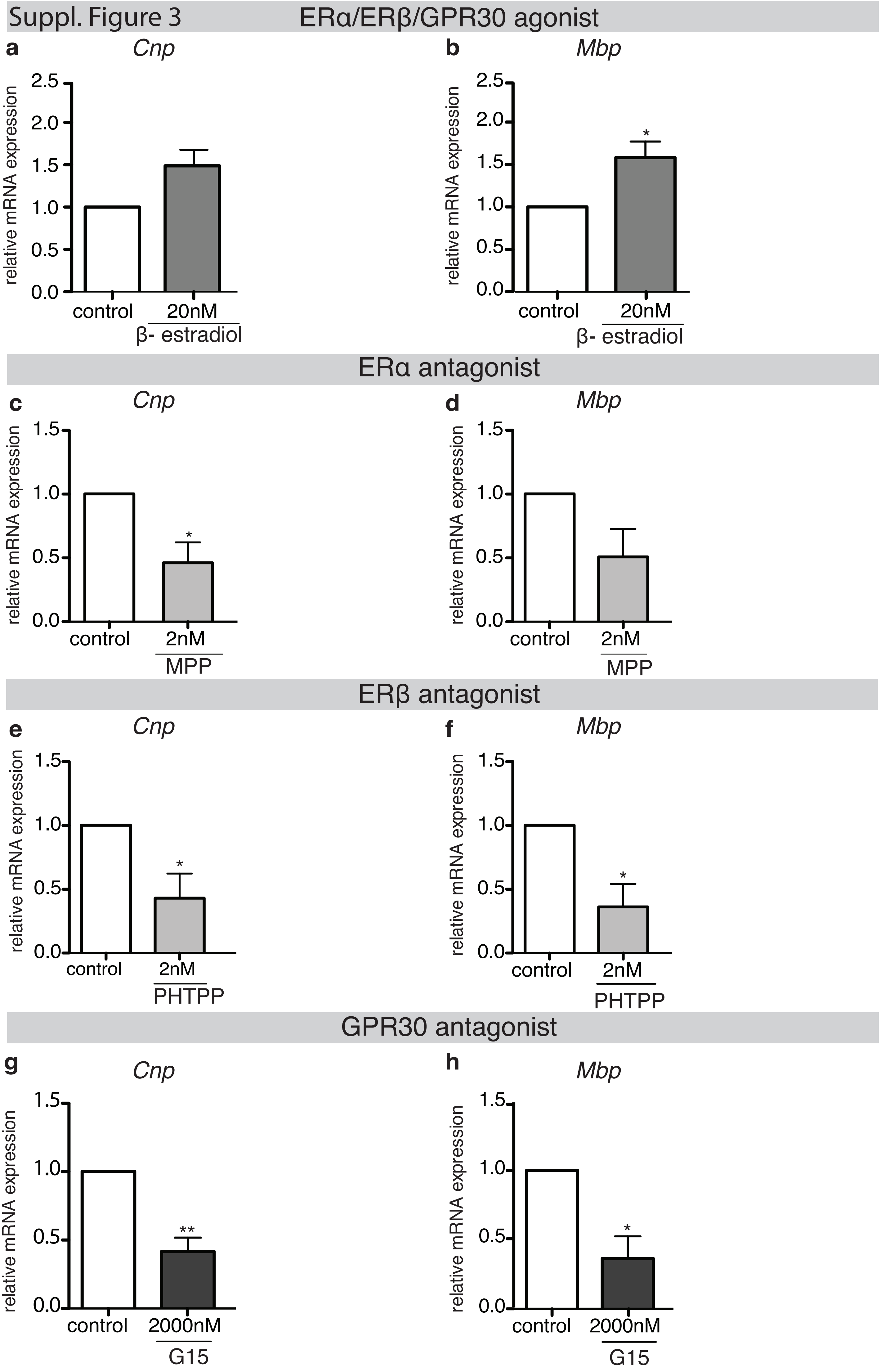

Supplemetary Fig-4

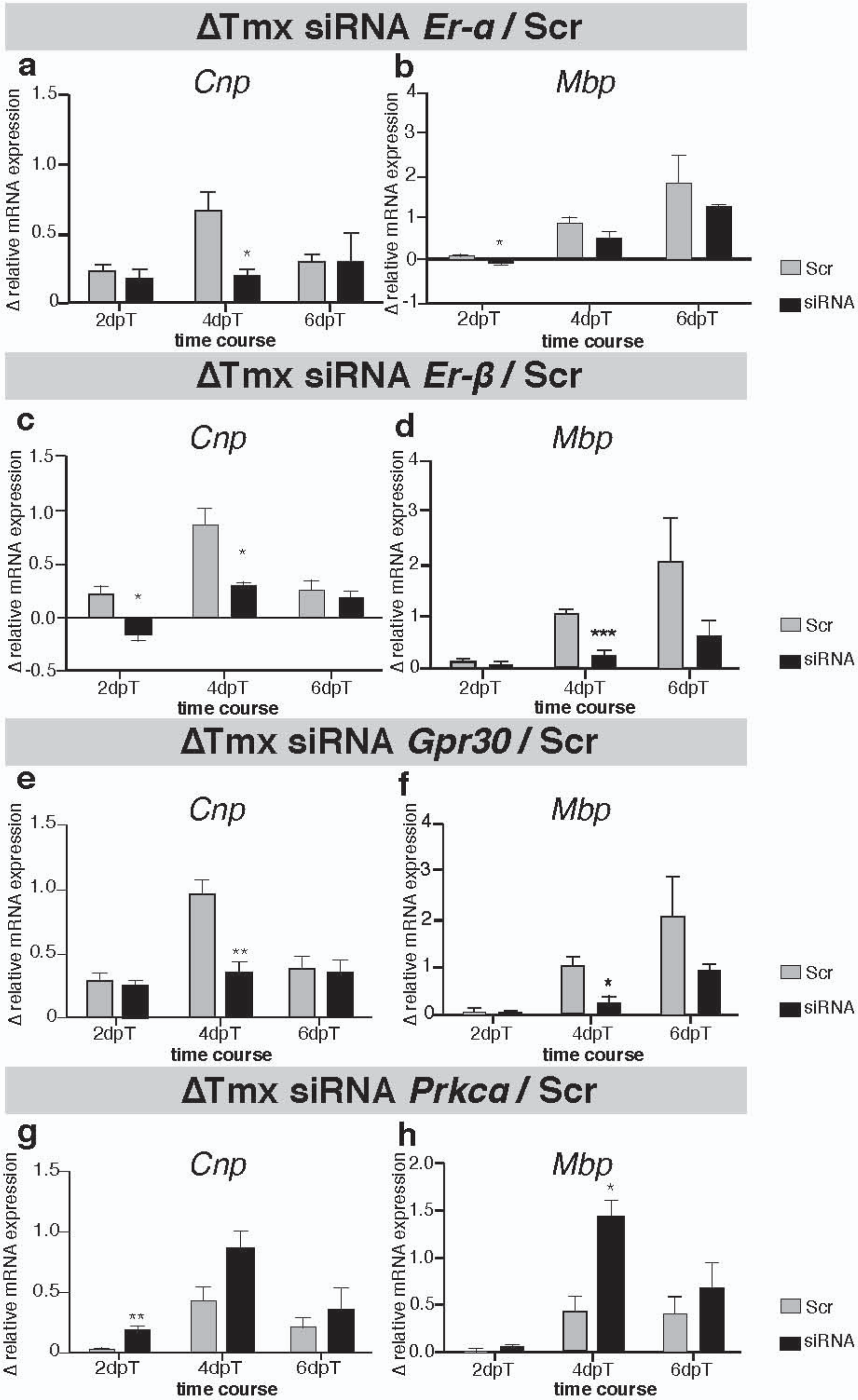

Suppl. Figure 5 (Kotter)

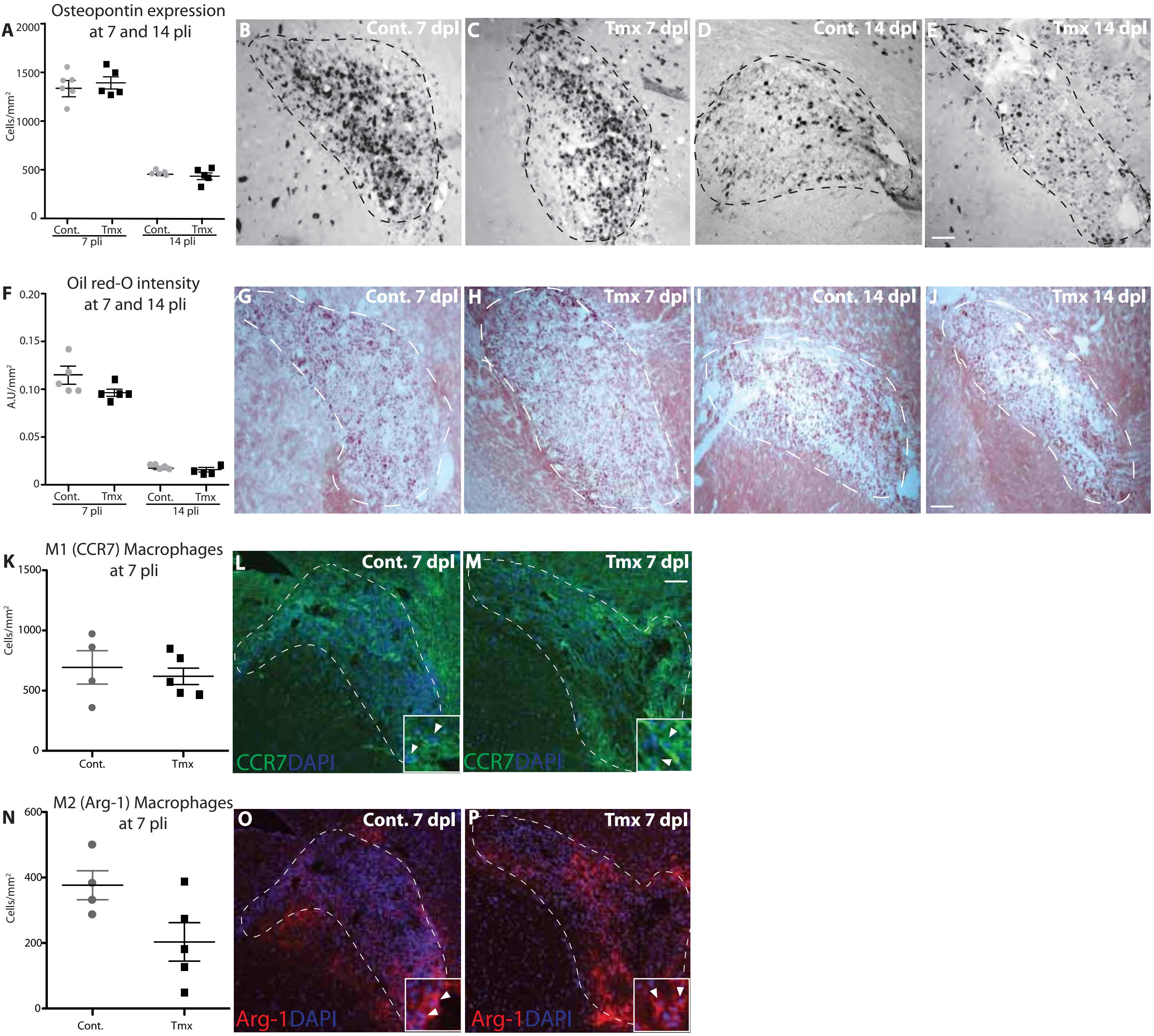

Supplement: Supplementary Information [file srep31599-s1.pdf]
